# Collagens VI and XII form complexes mediating osteoblast interactions during osteogenesis

**DOI:** 10.1007/s00441-015-2345-y

**Published:** 2016-01-12

**Authors:** Yayoi Izu, Yoichi Ezura, Manuel Koch, David E. Birk, Masaki Noda

**Affiliations:** Department of Molecular Pharmacology, Medical Research Institute, Tokyo Medical and Dental University, M&D Tower 24th, 5-45 1-Chome Yushima, Bunkyo-ku, Tokyo, 113-8549 Japan; Institute for Dental Research and Musculoskeletal Biology, Center for Biochemistry, University of Cologne, Cologne, Germany; Department of Molecular Pharmacology & Physiology, University of South Florida, Morsani College of Medicine, Tampa, Florida USA

**Keywords:** Collagen VI, Collagen XII, Communicating cell network, Osteogenesis, Primary osteoblasts

## Abstract

Bone formation is precisely regulated by cell-cell communication in osteoblasts. We have previously demonstrated that genetic deletion of *Col6a1* or *Col12a1* impairs osteoblast connections and/or communication in mice, resulting in bone mass reduction and bone fragility. Mutations of the genes encoding collagen VI cause Ullrich congenital muscular dystrophy (UCMD) and Bethlem myopathy (BM), which have overlapping phenotypes involving connective tissue and muscle. Recent studies have identified *COL12A1* gene mutations in patients with UCMD- and BM-like disorders harboring no *COL6* mutations, indicating the shared functions of these collagens in connective tissue homeostasis. The purpose of this investigation has been to test the hypothesis that collagens VI and XII have coordinate regulatory role(s) during bone formation. We analyzed the localization of collagens VI and XII relative to primary osteoblasts during osteogenesis. Immunofluorescence analysis demonstrated that collagens VI and XII colocalized in matrix bridges between adjacent cells during periods when osteoblasts were establishing cell-cell connections. Quantification of cells harboring collagen bridges demonstrated that matrix bridges were composed of collagens VI and XII but not collagen I. Interestingly, matrix bridge formation was impaired in osteoblasts deficient in either *Col6a1* or *Col12a1*, suggesting that both collagens were indispensable for matrix bridge formation. These data demonstrate, for the first time, a functional relationship between collagens VI and XII during osteogenesis and indicate that a complex containing collagens VI and XII is essential for the formation of a communicating cellular network during bone formation.

**Electronic supplementary material:**

The online version of this article (doi:10.1007/s00441-015-2345-y) contains supplementary material, which is available to authorized users.

## Introduction

Bone formation is precisely regulated by cell-cell communication in osteoblasts at bone-forming sites. During bone formation, pre-osteoblasts migrate toward the bone-forming sites where osteoblasts establish connections with adjacent osteoblasts, thereby forming a communicating cell network. This network allows osteoblasts to form appropriate amounts of high-quality bone. However, the mechanisms regulating this communicating cell network are still not clear.

Cells are surrounded by extracellular matrix in vivo; this matrix provides the proper environment for cells and varies depending on specific cellular events. We have previously demonstrated that collagens VI and XII are localized at bone-forming sites and that genetic deletion of *Col6a1* or *Col12a1* causes impaired osteoblast arrangement, resulting in decreased bone mass and strength (Izu et al. 2011b, 2012). In addition, osteoblast cellular events, such as polarization, which is required for osteoblast terminal maturation, bone matrix secretion, and cell-cell connection/communication via gap junctions, are impaired in *Col12a1*-deficient mice (Izu et al. 2011b). These data suggest that collagens VI and XII mediate cell communication during bone formation. However, the possible coordinated role(s) of collagens VI and XII in the regulation of osteoblast cell communication at bone-forming sites have not been defined.

Mutations in genes encoding collagen VI (i.e., *COL6A1*, *COL6A2*, and *COL6A3*) have been identified as causative in Ullrich congenital muscular dystrophy (UCMD) and Bethlem myopathy (BM). These diseases have overlapping phenotypes involving connective tissue and skeletal muscle. Both are early onset diseases, patients with UCMD have severe muscle weakness and distal joint hyperlaxity with proximal joint contractures, whereas in BM there is a relatively mild proximal weakness and distal joint contractures. However, not all patients with a clinical diagnosis of UCMD or BM have mutations in *COL6* genes. Recently, *COL12A1* gene mutations have been identified in patients with UCMD-like (Zou et al. 2014) and BM-like disorders (Hicks et al. 2014) without *COL6* mutations. Moreover, collagen XII deficiency has also been shown to contribute to UCMD- and BM-like phenotypes, as demonstrated by genetic deletion of *Col12a1* in mice, which results in muscular dystrophy, decreased grip strength (Zou et al. 2014), and connective tissue defects, such as kyphosis and decreased bone mass (Izu et al. 2011b). This supports the hypothesis that there is a mechanism(s) involving coordinated collagen VI and XII interactions in muscle and connective tissue development.

Collagen VI is a non-fibrillar collagen, forms characteristic microfibrillar networks, and is ubiquitously localized in connective tissues, including bone. The assembly of collagen VI is a multistep process; a short triple helical monomer consisting of α1(VI), α2(VI), and α3(VI) is formed and subsequently assembles into disulfide bonded antiparallel dimers. The dimers further assemble into tetramers (Allamand et al. 2011; Baldock et al. 2003; Ball et al. 2003; Engel et al. 1985; Engvall et al. 1986; Mienaltowski and Birk. 2014). Collagen VI is secreted as a tetramer, which forms microfibril networks in the extracellular milieu. Collagen XII is also a non-fibrillar collagen and is widely expressed in connective tissues, including bone, ligaments, tendons, fibrocartilage, smooth muscle, skin (Walchli et al. 1994), articular cartilage (Watt et al. 1992), and cornea (Anderson et al. 2000; Hemmavanh et al. 2013). In contrast to collagen VI, collagen XII belongs to the family of fibril-associated collagens with interrupted triple helices (FACIT; Chiquet et al. 2014; Dublet et al. 1989; Gordon et al. 1987; Oh et al. 1992) and consists of a homotrimer of α1(XII) chains at the C-terminus with three non-collagenous domains and a large globular N-terminal domain. Therefore, these collagens are structurally distinct; however, mutations in both collagen genes cause common diseases.

Collagen VI interacts with a wide variety of proteins via its globular domain, which contains numerous different binding sites (Chen et al. 2015; Doane et al. 1998; Howell and Doane. 1998). On the other hand, collagen XII interacts with collagen I via the collagenous domain (Font et al. 1996; Keene et al. 1991; Koch et al. 1995; Nishiyama et al. 1994), and a large N-terminal globular domain, NC3, provides a possible interaction with other molecules such as tenascin X (Veit et al. 2006), decorin, and fibromodulin (Font et al. 1996, 1998; Massoudi et al. 2012). Therefore, both collagens have the ability to mediate cell-matrix and matrix-matrix interactions, which are important features regulating cell migration, adhesion, apoptosis, and survival. Based on these shared functions, there may be a common regulatory system mediated by collagens VI and XII.

Here, we demonstrate that collagens VI and XII are spatially co-localized during osteoblast development in primary osteoblasts derived from neonatal mouse calvaria. This colocalization is restricted to matrix bridges that lie between adjacent cells and that are formed when osteoblasts make cell-cell connections. Since collagen I is virtually absent from matrix bridges and collagens VI and XII are indispensible for matrix bridge formation, we propose the existence of a collagen VI/XII complex that has a novel regulatory role(s) in mediating cell-cell interactions during communicating cell network formation at bone-forming sites.

## Materials and methods

### Cell culture

Primary osteoblasts were obtained from calvaria of wild-type, *Col6a1*^−/−^, or *Col12a*1^−/−^ neonatal mice by using sequential enzyme digestion, as previously described (Izu et al. 2011b). Primary osteoblasts were maintained in α-minimal essential medium supplemented with 10 % fetal bovine serum (FBS) and antibiotics (Life Technologies, Rockville, Md., USA) as a non-osteogenic medium. Non-osteogenic medium supplemented with phospho-ascorbic acid (100 μg/ml) and β-glycerophosphate (10 mM) was used as osteogenic medium to induce osteogenesis in primary osteoblasts that were previously maintained in non-osteogenic medium.

### Immmunolocalization analysis

Immunofluorescence analysis was performed in primary osteoblasts as previously described (Izu et al. 2011a, 2011b). Briefly, primary osteoblasts cultured in non-osteogenic medium were seeded on 18-mm cover slips at 2 × 10^4^ cells/well in a 12-well plate and further cultured in either non-osteogenic or osteogenic medium. Cells were then fixed with 4 % paraformaldehyde on days 2, 4 (confluent), and 11 (7 days after confluence). The coverslips were blocked with 10 % goat serum with or without prior permeabilization with 0.3 % Triton X-100. Samples were then incubated with rabbit polyclonal anti-collagen VI (1:500; Cosmo Bio, Tokyo, Japan), rabbit polyclonal anti-collagen XII (1:250; Izu et al. 2011b), guinea pig polyclonal anti-collagen XII (1:250; Izu et al. 2011b), and/or rabbit polyclonal anti-collagen I (1:500, Life Technologies) at 4 °C overnight. Secondary antibodies (goat anti-rabbit IgG Alexa 488 or goat anti-guinea pig IgG Alexa 546) were used at a 1:500 dilution. As a negative control, cells were incubated with secondary antibodies only. In addition, cells were labeled with vibrant DiI (1,1′-dioctadecyl 3,3,3′,3′-tetramethylindocarbocyanine perchlorate; 1:200; Thermo Fisher Scientific, Mass., USA), stained with Alexa-488-labeled phalloidin (1:200; Life Technologies), or imaged as phase contrast to visualize cell shapes. Prolong Gold DAPI (4,6-diamidino-2-phenylindole) mounting medium (Life Technologies) was used for nuclear localization. Images were captured by using a confocal laser-scanning microscope (Fluo-View FV10i; Olympus, Tokyo, Japan) or a fluorescence microscope (Olympus BX51; Olympus).

### Western blot analysis

Primary osteoblasts cultured for 2, 4, or 11 days were used for western blot analysis. Medium was changed to non-osteogenic or osteogenic medium without FBS 1 day before harvesting. Media and cells, which were lysed in lysis buffer containing 50 mM TRIS–HCl, 1 M NaCl, 10 mM EDTA, and 0.1 % SDS, were used for SDS-polyacrylamide gel electrophoresis (SDS-PAGE) and western blotting. SDS-PAGE was performed with 4–15 % acrylamide gel under denaturing conditions. Western blotting was performed as previously described (Izu et al. 2009) by using rabbit polyclonal anti-collagen VI (1:1000; Novus Biologicals, Littleton, Colo., USA), rabbit polyclonal anti-collagen XII (1:1000; Izu et al. 2011b), rabbit polyclonal anti-collagen I (1:3000; Life Technologies), and mouse anti-β-actin (1:4000; Sigma, St Louis, Mo., USA) primary antibodies and anti-mouse or anti-rabbit horseradish peroxidase (HRP)-conjugated secondary antibodies (Jackson ImmunoResearch, Westgrove, Pa., USA).

### Quantitative analysis of cells connected via collagen bridges

The numbers of cells connected through collagens were analyzed. Ten digital images (magnification: 20×) were randomly selected from cells immunostained for collagens I, VI, or XII with DiI labeling on days 2 and 4. The total number of cells was counted by using DiI labeling to exclude cells lacking a part of the cell body. Among the total cells, cells which had collagen bridges were counted. The cell count was performed in triplicate from each immunostaining group and is given as the mean of triplicate determinants. Statistical analysis was performed by using Student’s *t*-test.

## Results

### Collagen VI links adjacent primary osteoblasts

**Fig. 1:**
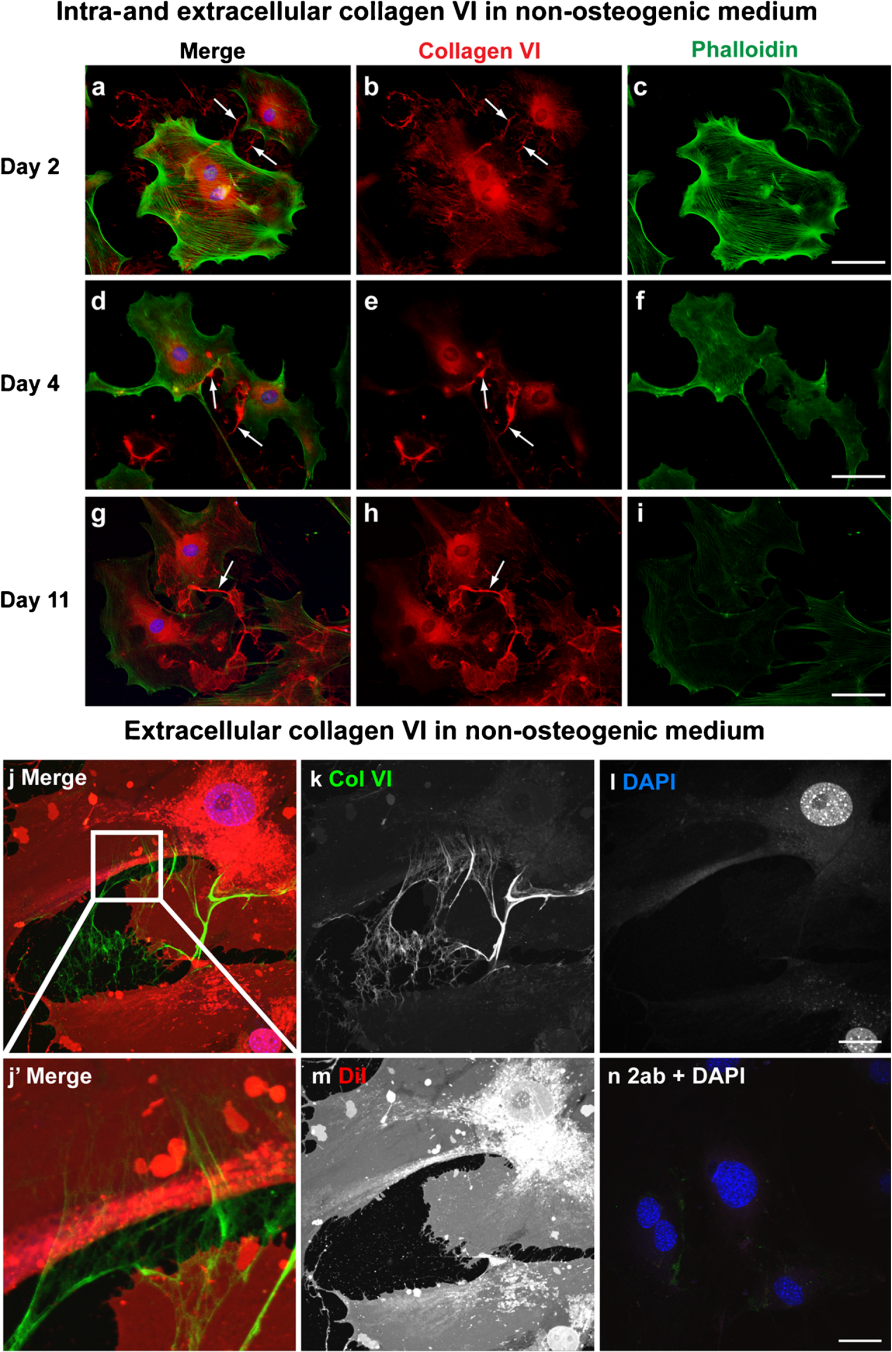
Collagen VI localization in primary osteoblasts cultured in non-osteogenic medium. Immunofluorescence analysis of collagen VI was performed in primary osteoblasts under non-osteogenic conditions. The cells were harvested on days 2 (**a-c**), 4 (**d-f**), and 11 (**g-i**) and immunostained for collagen VI (*red*) with permeabilization by Triton X-100. Phalloidin (*green*) and DAPI (*blue*) were used to detect cell shape and nuclei, respectively. As shown in the merged images, collagen VI immunoreactivity was observed intra- and extracellularly. Extracellular collagen VI was localized between adjacent osteoblasts (*arrows*) on day 2 (**a**, **b**). This extracellular localization was gradually increased on days 4 (**d**, **e**) and 11 (**g**, **h**). *Bars* 50 μm. On day 4, osteoblasts were stained for collagen VI (*green*) without permeabilization and then captured by confocal microscopy (**j-n**). DiI (*red*) and DAPI (*blue*) were used to detect cell shape and nuclei, respectively (*2ab* secondary antibody only as a control (**n**)). As shown in the high magnification image in the *inset* from the merged image (**j’**), extracellular collagen VI connected cell bodies without the presence of protrusions. *Bars* 25 μm

**Fig. 2:**
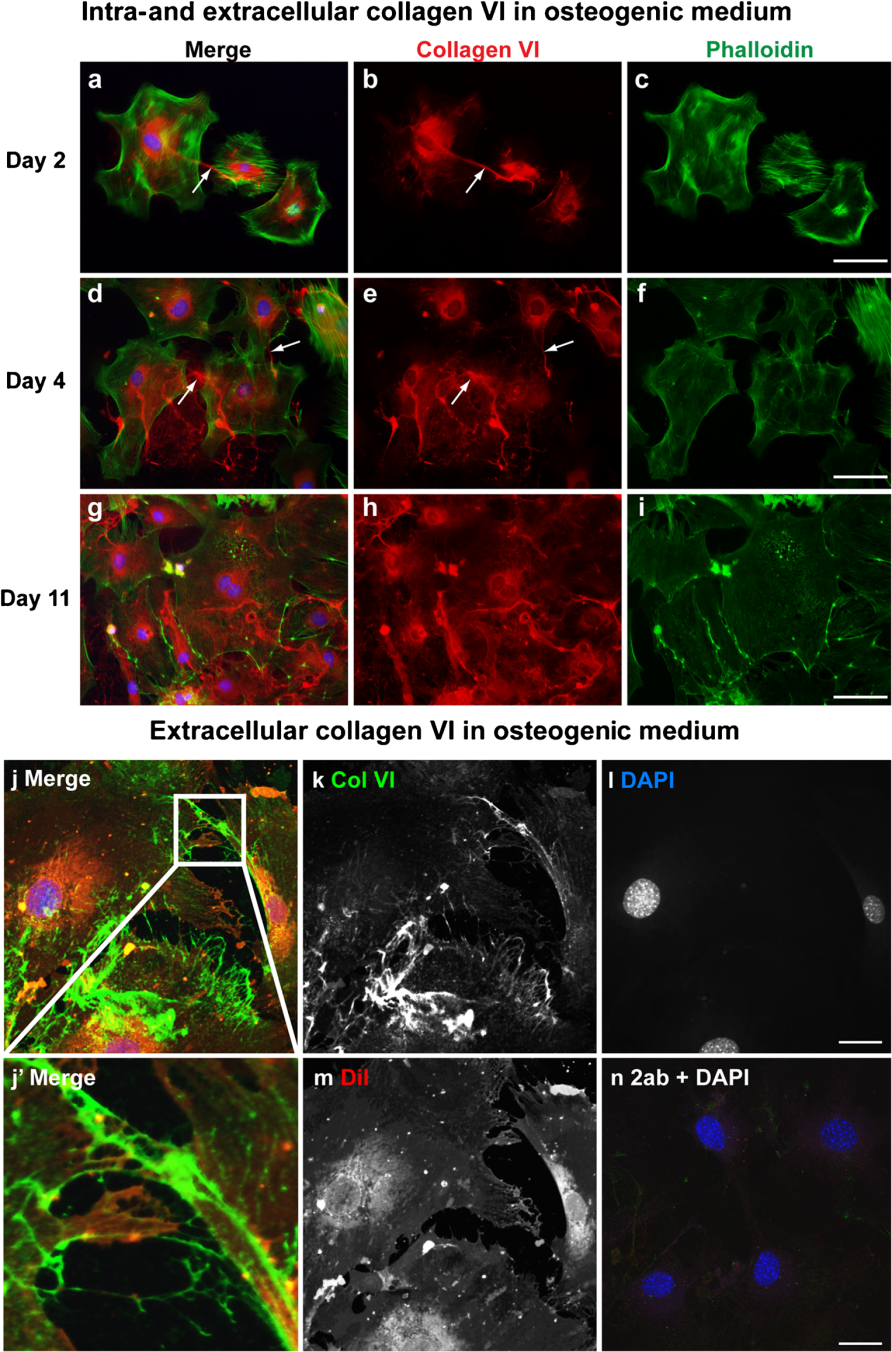
Collagen VI localization in matrix bridges connecting adjacent cells in osteogenic medium. Immunofluorescence analysis of collagen VI was performed in primary osteoblasts in osteogenic medium. The cells were harvested on days 2 (**a-c**), 4 (**d-f**), and 11 (**g-i**) and immunostained for collagen VI (*red*) with permeabilization by Triton X-100. Phalloidin (*green*) and DAPI (*blue*) were used to detect cell shape and nuclei, respectively. As shown in the merged image, collagen VI immunoreactivity was observed intra- and extracellularly. Extracellular collagen VI was localized between adjacent osteoblasts (*arrows*) on day 2 (**a**, **b**). This extracellular localization was gradually increased on days 4 (**d**, **e**) and 11 (**g**, **h**). Osteogenic medium increased collagen VI expression when compared to non-osteogenic medium. *Bars* 50 μm. On day 4, osteoblasts were stained for collagen XII (*green*) without permeabilization and then captured by confocal microscopy (**j-n**). The merged image demonstrates that extracellular collagen VI is localized between adjacent cells (*2ab* secondary antibody only as a control (**n**)). The high magnification image in the *inset* from the merged image reveals that extracellular collagen VI connects cell bodies without the presence of protrusions (**j’**). *Bars* 25 μm

To analyze collagen VI localization during osteogenesis, collagen VI immunofluorescence was performed with permeabilization by using primary osteoblasts obtained from neonatal mouse calvaria. Osteoblasts were cultured in non-osteogenic medium (Fig. 1) or osteogenic medium (Fig. 2) and were harvested on days 2, 4, and 11. In non-osteogenic medium, collagen VI was localized mainly in the cytoplasm, with some extracellular extensions directed toward adjacent cells on day 2 (Fig. 1a-c). The extracellular localization of collagen VI between adjacent osteoblasts was detected as matrix bridges, and the number of matrix bridges was increased on day 4 (Fig. 1d-f). On day 11, extracellular localization of collagen VI was increased throughout the culture thereby masking the collagen VI bridge structure (Fig. 1g-i).

To determine whether collagen VI bridges were localized in the intra- or extracellular milieu, cell shape was visualized by using DiI (a lipophilic membrane stain) and immunostaining of collagen VI without permeabilization, allowing visualization of extracellular collagen VI only. Cells from 4-day cultures in non-osteogenic medium were analyzed using confocal microscopy (Fig. 1j-n), revealing fine structures containing collagen VI. These structures formed adjacent to the cell surface and extended toward adjacent cells, resulting in the formation of matrix bridges.

Similar to our observations in non-osteogenic medium, osteogenic medium induced collagen VI matrix bridge formation and intracellular localization (Fig. 2a-i). Extracellular collagen VI was increased in osteogenic medium, compared with that in non-osteogenic medium at all stages analyzed. On days 2 and 4, when cells begin to form communicating cell networks, extracellular collagen VI was found to be expressed as matrix bridges, connecting adjacent cells (Fig. 2a-f). On day 11, extracellular collagen VI was visualized as well-organized microfiber networks surrounding individual cells (Fig. 2g-i). The extracellular localization of collagen VI in primary osteoblasts on day 4 in osteogenic medium was analyzed by confocal microscopy without permeabilization (Fig. 2j-n). Extracellular collagen VI microfibers accumulated and became thicker in matrix bridges (Fig. 2j). Therefore, collagen VI may play a role in cell-cell interactions by accumulating in matrix bridges between adjacent cells beginning at early stages of osteogenesis.

### Extracellular collagen XII bridges adjacent cells in primary osteoblasts during osteogenesis

**Fig. 3:**
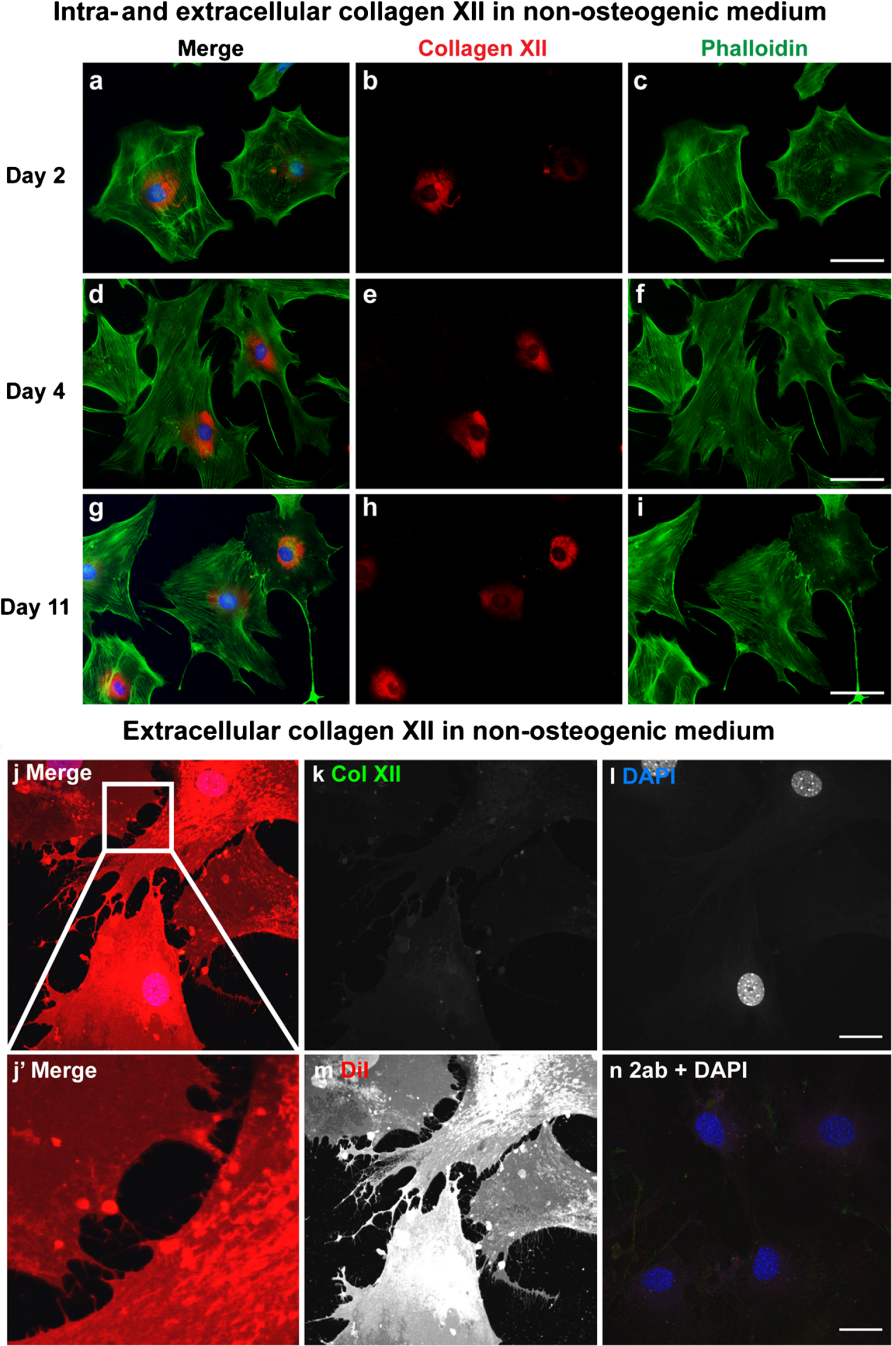
Collagen XII is not localized extracellularly in non-osteogenic medium. Immunofluorescence analysis of collagen XII was performed in primary osteoblasts in non-osteogenic medium. The cells were harvested on days 2 (**a-c**), 4 (**d-f**), and 11 (**g-i**) and immunostained for collagen XII (*red*) with permeabilization. Phalloidin (*green*) and DAPI (*blue*) were used to detect cell shape and nuclei, respectively. Immunoreactivity for collagen XII was observed intracellularly but not extracellularly at all the stages. *Bars* 50 μm. On day 4, osteoblasts were stained for collagen XII (*green*) without permeabilization and then captured by confocal microscopy (**j-n**). DiI (*red*) and DAPI (*blue*) were used to detect cell shape and nuclei, respectively (*2ab* secondary antibody only as a control). As shown in the merged image, no immunoreactivity was detected for collagen XII. The high magnification image in the *inset* from the merged image reveals no collagen XII immunoreactivity between adjacent cells connected by cell protrusions, as shown by DiI staining (**j’**). *Bars* 25 μm

**Fig. 4:**
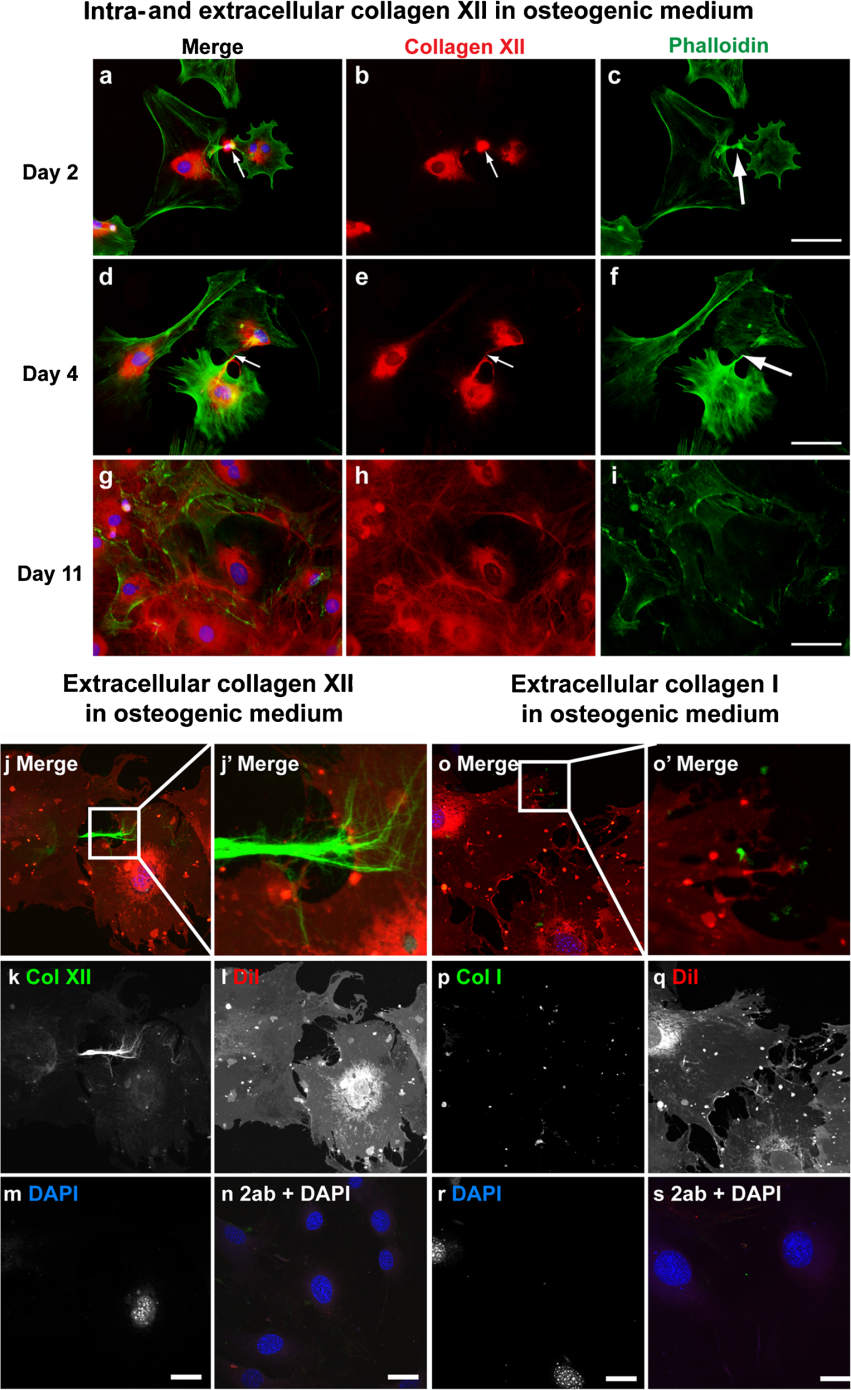
Extracellular collagen XII, but not collagen I, forms matrix bridges under osteogenic conditions. Immunofluorescence analysis of collagen XII (**a-n**) and collagen I (**o-s**) in primary osteoblasts cultured in osteogenic medium. The cells were harvested on days 2 (**a-c**), 4 (**d-f**), and 11 (**g-i**) and immunostained for collagen XII (*red*) with permeabilization. Phalloidin (*green*) and DAPI (*blue*) were used to detect cell shape and nuclei, respectively. Both intra- and extracellular immunoreactivity for collagen XII was detected. Extracellular collagen XII was localized between adjacent cells (*arrows*) on days 2 (**a**, **b**) and 4 (**d**, **e**). On day 11, collagen XII was detected as extracellular microfibril networks surrounding individual cells (**g**, **h**). *Bars* 50 μm. Confocal analysis reveals collagen XII (*green*) immunofluorescence without permeabilization in osteoblasts on day 4 (**j-n**). DiI (*red*) and DAPI (*blue*) were used to detect cell shape and nuclei, respectively (*2ab* secondary antibody only as a control). Extracellular microfibers of collagen XII were localized between adjacent cells and connected each cell (**j**). As seen in the high magnification image in the *inset*, distinct collagen XII matrix bridges were attached to the adjacent cell (**j’**). No cell protrusions, as shown by DiI, were observed with the matrix bridges. *Bars* 25 μm. Confocal images of collagen I (*green*) in primary osteoblasts cultured in osteogenic medium for 4 days, staining without permeabilization (**o-s**). DiI (*red*) and DAPI (*blue*) were used to detect cell shape and nuclei, respectively (*2ab* secondary antibody only as a control). Extracellular collagen I was localized pericellularly (**p**). As shown in the high magnification image in the *inset*, extracellular collagen I was localized on the cell surface, and no matrix bridges were detected (**o’**). *Bars* 25 μm

To elucidate collagen XII localization during osteogenesis, collagen XII immunostaining with permeabilization was carried out in primary osteoblasts cultured under non-osteogenic (Fig. 3) or osteogenic (Fig. 4) conditions. Collagen XII localization was restricted to the cytoplasm in non-osteogenic medium (Fig. 3a-i). This was confirmed by the lack of extracellular collagen XII when the cells were immunostained without permeabilization (Fig. 3j-n). In contrast, collagen XII was detected in the intra- and extracellular milieu when cells were cultured in osteogenic medium (Fig. 4a-n). Extracellular localization of collagen XII was detected in matrix bridges on days 2 and 4 in osteogenic medium (Fig. 4a-f). By day 11, collagen XII was detected as an extracellular microfiber network (Fig. 4g-i). Detailed analysis of extracellular collagen XII localization on day 4 was performed in osteoblasts cultured under osteogenic condition without permeabilization using confocal microscopy (Fig. 4j-n). The data demonstrate that extracellular collagen XII indeed forms matrix bridges connecting adjacent osteoblasts.

To elucidate whether collagen matrix bridges were specific for collagens VI and XII, we next investigated the localization of collagen I by confocal microscopy in primary osteoblasts cultured in osteogenic medium (Fig. 4o-s). In contrast to the observed localization of collagens VI and XII, extracellular collagen I was localized pericellularly, suggesting that collagen I does not form matrix bridges under conditions in which matrix bridges were formed by collagens VI and XII.

### Collagens VI and XII colocalize during formation of communicating cell networks under osteogenic conditions

**Fig. 5:**
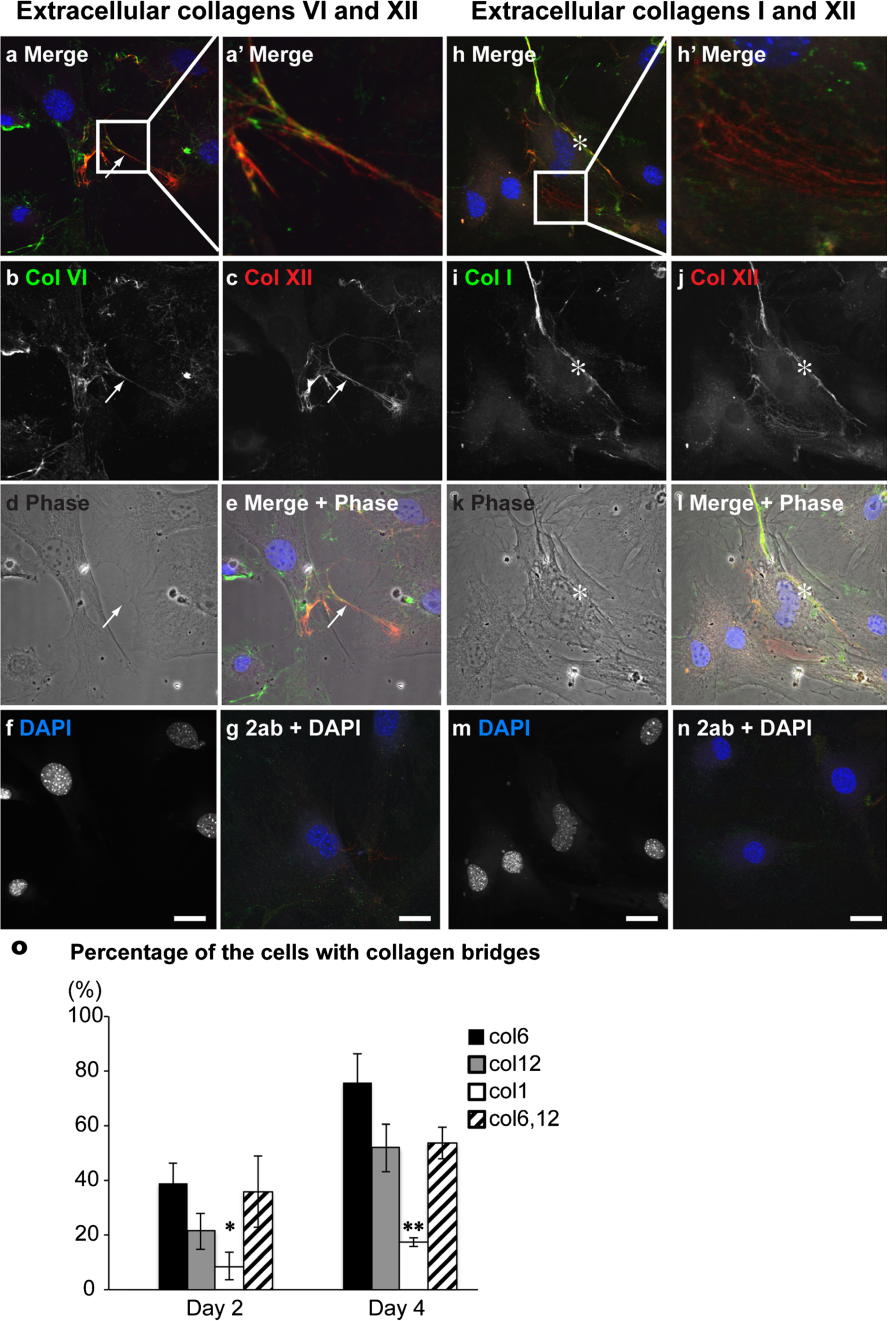
Matrix bridge formation is specific for collagens VI and XII, but not collagen I under osteogenic conditions. Confocal images of double immunostaining for collagens VI and XII (**a-g**) and collagens I and XII (**h-n**) in primary osteoblasts cultured for 4 days under osteogenic conditions without permeabilization. DAPI (*blue*) and phase contrast (*gray*) were used to detect nuclear localization and cell shape, respectively. Osteoblasts stained with secondary antibodies and DAPI were used as a negative control (*2ab + DAPI*). The merged image demonstrates the localization of collagens VI (*green*) and XII (*red*) in a matrix bridge between adjacent cells (*arrows* in **a-e**). The high magnification image in the *inset* demonstrates partial colocalization of collagens VI and XII (**a’**). As shown in the merged image, colocalization of collagens I (*green*) and XII (*red*) was detected pericellularly (*asterisks* in **h-l**). The high magnification image in the *inset* demonstrates collagen XII localization between adjacent cells but not collagen I (**h’**). *Bars* 25 μm. The number of cells connected via collagens VI, XII, and I was analyzed in immunofluorescence images after cells had been cultured in osteogenic medium for 2 and 4 days (**o**). The mean percentages of cells which had collagen bridges were calculated based on the total number of cells observed in each image. Ten digital images were used from cells immunostained for collagens I, VI, XII, or VI and XII. Measurements were performed in triplicate. The number of cells with collagen VI or XII bridges was higher than that with collagen I bridges on day 2. In addition, the percentage of cells which had both collagens VI and XII was significantly higher than that which had collagen I bridges. The number of cells with collagen VI, collagen XII, or both collagens VI and XII was increased, whereas that of cells with collagen I did not change on day 4. Statistical analysis revealed that the number of cells with collagen VI and/or collagen XII matrix bridges was significantly higher than that with collagen I on days 2 (*P < 0.06) and 4 (**P < 0.005)

To investigate whether collagens VI and XII were colocalized in matrix bridges, double immunostaining for collagens VI and XII was performed in primary osteoblasts under non-osteogenic (Supplementary Fig. 1a) or osteogenic (Fig. 5a-g, Supplementary Fig. 1b) conditions. Under non-osteogenic conditions, only collagen VI was detected in the extracellular milieu, as expected (Supplementary Fig. 1a). In contrast, although collagen VI immunoreactivity exhibited a broader localization than that of collagen XII (Supplementary Fig. 1), both collagens were localized in matrix bridges, where they were partially colocalized on days 2 and 4. Confocal images with phase contrast clearly demonstrated the presence of extracellular collagens VI and XII in matrix bridges, with partial colocalization observed on day 4 (Fig. 5a-g). These observations indicate that collagens VI and XII coordinately regulate the formation of communicating cell networks during osteogenesis.

### Collagens I and XII do not interact during matrix bridge formation

Collagens I and XII are thought to interact during fibrillogenesis (Font et al. 1996; Keene et al. 1991; Koch et al. 1995; Nishiyama et al. 1994). Therefore, we next analyzed the localization of collagens I and XII during matrix bridge formation under non-osteogenic (Supplementary Fig. 2a) or osteogenic (Fig. 5h-n, Supplementary Fig. 2b) conditions. As expected, no extracellular localization of collagens I and XII was detected under non-osteogenic conditions (Supplementary Fig. 2a). Although collagens I and XII were colocalized close to the cell surface on days 2 and 4 in osteogenic medium, no colocalization in matrix bridges was detected. On day 11, no clear matrix bridge formation was detected because collagens I and XII were both observed in the extracellular milieu forming microfiber networks. A detailed analysis by confocal microscopy in primary osteoblasts revealed that collagens I and XII were colocalized pericellularly but not in matrix bridges in which only collagen XII immunoreactivity was observed (Fig. 5h). These data suggest that collagens I and XII do not interact during matrix bridge formation.

To support these findings further, western blot analysis was performed in primary osteoblasts cultured in non-osteogenic or osteogenic medium (Supplementary Fig. 3). Consistent with the immunostaining data, collagens VI, XII, and I were expressed in cells cultured under both non-osteogenic and osteogenic conditions. Collagen VI expression in culture medium was similar between non-osteogenic and osteogenic conditions, whereas collagen XII expression in culture medium was only observed under osteogenic conditions, suggesting that the extracellular localization of collagen XII only occurred when cells were cultured in osteogenic medium. Similar to collagen XII, the secreted form of collagen I, which was visualized as the lower band, was only detected in osteogenic medium.

### Cells are bridged via collagens VI and XII, but not collagen I

We next quantified matrix bridge formation by counting cells harboring collagen bridges (Fig. 5o). Cells were counted based on the immunofluorescence images of cells cultured under osteogenic conditions. The percentages of the cells with collagen VI bridges on days 2 and 4 were 39 % and 75 %, respectively. Collagen XII bridges were observed in 21 % of cells on day 2 and 51 % of cells on day 4. By contrast, the percentages of cells harboring collagen I on days 2 and 4 were 8 % and 17 %, respectively. We also analyzed the percentage of cells which had bridges positive for both collagens VI and XII. On days 2 and 4, the percentages were 35 % and 53 %, respectively, similar to the data obtained for the separate analyses of collagens VI or XII. Statistical analysis revealed that the percentages of cells with collagens VI and XII were significantly higher than those with collagen I. Therefore, the matrix bridges formed in adjacent osteoblasts consist of collagens VI and XII.

### Complex containing collagens VI and XII is required for matrix bridge formation during osteogenesis

**Fig. 6:**
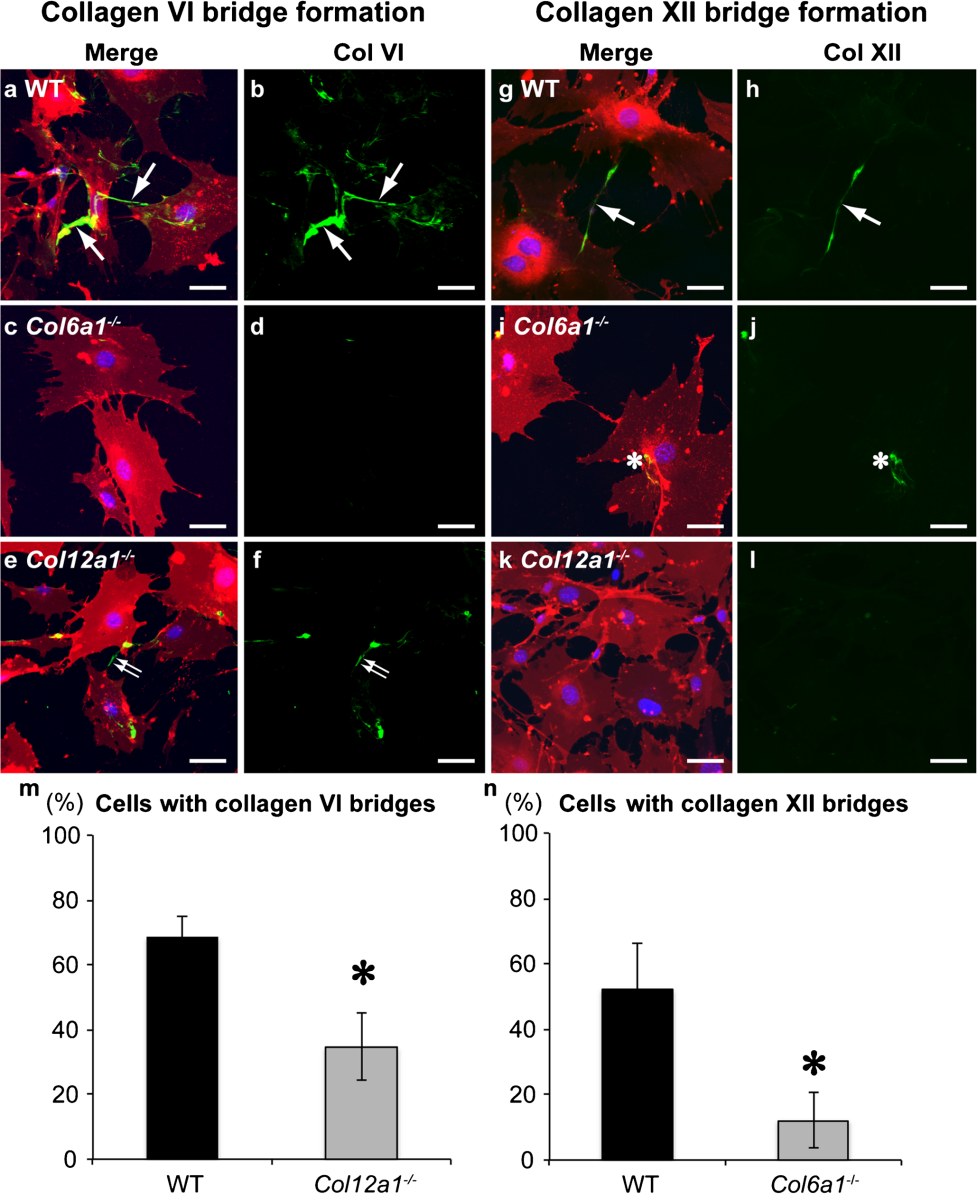
Collagen VI or XII deficiency disrupts matrix bridge formation. Primary osteoblasts obtained from wild-type (*WT*), *Col6a1* ^−/−^, or *Col12a1* ^−/−^ mice were cultured in osteogenic medium for 4 days and were then immunostained for collagen VI (**a-f**) and XII (**g-l**) with DiI (*red*) and DAPI (*blue*). Collagen VI (*green*) bridge formation (*arrows*) between adjacent cells was detected in osteoblasts from WT mice (**a**, **b**). In contrast, most collagen VI was accumulated on the cell surface, and a few fine bridges (*double arrows*) were detected in *Col12a1* ^*−/−*^ osteoblasts (**e**, **f**). Similar to collagen VI staining in *Col12a1* ^*−/−*^ osteoblasts, collagen XII (*green*) expression was limited in *Col6a1* ^*−/−*^ osteoblasts (*asterisks* in **i**, **j**). Note the collagen XII matrix bridge (*arrow*) **g**, **h**. *Bars* 50 μm Quantification of collagen bridges (**m**, **n**). The percentage of cells harboring collagen bridges was calculated based on immunostaining on day 4. Collagen VI bridge formation was significantly decreased in *Col12a1* ^*−/−*^ osteoblasts compared with WT osteoblasts (**m**). Similarly, collagen XII bridge formation was significantly decreased in *Col6a1* ^*−/−*^ osteoblasts (**n**). *P < 0.02

To elucidate the possible functional relationship between collagens VI and XII, we next investigated whether deficiencies in collagen VI or XII affect matrix bridge formation during osteogenesis. Immunostaining for collagen VI or XII was performed in the primary osteoblasts obtained from wild-type, *Col6a1*^−/−^, and *Col12a1*^−/−^ mice after culture under osteogenic conditions (Fig. 6a-l). Matrix bridge formation was absent or decreased in *Col6a1*^−/−^ or *Col12a1*^−/−^ osteoblasts (Fig. 6a-l). Quantification of cells which had matrix bridges revealed that matrix bridge formation was significantly decreased in primary osteoblasts from *Col6a1*^−/−^ or *Col12a1*^−/−^ mice compared with that from wild-type mice (Fig. 6m, n). These data strongly indicate that collagens VI and XII form a complex during matrix bridge formation, and that this complex is required in order to establish communicating cell networks during bone formation.

## Discussion

During bone formation, establishment of well-organized communicating cell networks is essential for appropriate osteoblast maturation and subsequent bone matrix secretion, which collectively define bone volume and quality. Here, we demonstrate, for the first time, the existence of a novel coordinated regulatory mechanism involving collagens VI and XII. This regulates the formation of communicating osteoblast networks that control/promote bone formation. Matrix bridge formation is specific for collagens VI and XII, and the regulation of this process requires the presence of both collagens. Our data indicate that the interaction between collagens VI and XII regulates cell-cell interactions at bone-forming sites during bone formation.

At bone-forming sites, well-arranged osteoblasts interact through gap junctions, tight junctions, and the receptor-ligand system. Impaired cell-cell interactions result in defects in bone mass (Chaible et al. 2011; Lecanda et al. 2000; Watkins et al. 2011); therefore, cell-cell interactions are essential for the formation of healthy, strong bones. Our present data indicate that collagens VI and XII colocalize, and that this colocalization is restricted to matrix bridges between adjacent osteoblasts during the establishment of communicating cell networks. Interestingly, we also found that collagens XII and I colocalize; however, the localization pattern of these collagens is distinct from that observed for collagens VI and XII. Thus, the function of the collagen VI and XII complex is as a specific regulator, facilitating communicating cell network formation that is required at the beginning of bone formation.

In addition to the colocalization of collagens VI and XII, we also demonstrate that deficiencies in collagen VI or XII impair matrix bridge formation, suggesting that matrix bridge formation requires both collagens VI and XII. This is the first evidence demonstrating a functional interaction between collagens VI and XII. In support of the current data, our previous reports demonstrate that deficiencies in collagen VI (Izu et al. 2012) and collagen XII (Izu et al. 2011b) result in a disorganized osteoblast arrangement at bone-forming sites and therefore cause decreased bone mass and fragility. We have also demonstrated that collagen XII regulates cell-cell communications via gap junctions in osteoblasts. These observations strongly indicate that the collagen VI/XII complex regulates the sequence of events necessary for the formation of communicating cell networks. Therefore, these interactions are critical in establishing well-organized osteoblasts at bone-forming sites.

The structure of collagen matrix bridges, as determined in this study, might be similar to that of cell protrusions or cell bridges (Zhang and Zhang 2013). Our immunolocalization analysis has occasionally demonstrated collagen VI and XII localization on the tips of cell protrusions. However, most collagen VI and XII bridges seem to connect cell bodies rather than cell protrusions. In addition, immunostaining without the use of permeabilization together with DiI labeling or phase contrast images has revealed the distinct extracellular localization of collagens VI and XII. In the typical process of fibril formation with collagen I, procollagens are released into the extracellular environment through secretory vacuoles known as Golgi-to-plasma membrane carriers (GPCs). Although the details of the GPC secretion pathway are still under investigation, fibril formation is known to occur in compartments associated with both cell protrusions and cell bodies, and no clear evidence has demonstrated that protrusion formation is required for collagen secretion (Banos et al. 2008; Birk and Trelstad. 1986; Kalson et al. 2015). Further studies should investigate the mechanisms involved in matrix bridge formation.

In this study, we have found that collagen VI deficiency decreases collagen XII deposition in our culture system and vice versa. These data are inconsistent with a previous report in which no abnormalities in collagen VI immunoreactivity have been observed in fibroblasts obtained from patients with *COL12A1* mutations (Hicks et al. 2014), and the same is true for the *COL6* mutation cases. This can be explained by differences in the culture period, because we analyzed the cells as cell-cell connections are beginning to form, whereas skin fibroblasts in the previous study were analyzed at confluence (Hicks et al. 2014). Alternatively, this difference might be explained by differences in cell type (Jimenez-Mallebrera et al. 2006). Although the data are different to some degree, both studies support the idea that a deficiency in at least one type of collagen is sufficient to cause BM and UCMD. Therefore, the coordinated role(s) of collagens VI and XII in a single mechanism are indispensable for the function of the complex, and each collagen cannot compensate for the dysfunction of the other.

The comparison between non-osteogenic and osteogenic medium has revealed that the extracellular localization of collagen VI is independent of the osteogenic medium, whereas the extracellular collagens I and XII are restricted under osteogenic conditions. This is consistent with our western blot analysis of culture medium, suggesting that collagen VI is secreted prior to collagen I and XII by osteoblasts during very early stages. However, collagen VI assembly might not be sensitive to ascorbate, which is generally required for collagen triple helix formation, since no ascorbate was supplemented in the non-osteogenic medium. In contrast, triple helixes of collagens I and XII might not be stable and could be retained intracellularly without ascorbate (Franceschi and Iyer 1992).

Alternatively, the secretion of collagens I and XII might be closely associated. Collagen XII belongs to the FACIT subfamily and is associated with collagen I fibril assembly. In addition, our data have demonstrated the pericellular colocalization of collagens I and XII during osteogenesis. Many studies have shown that *Col1a1* mRNA is not expressed during very early stages of osteogenesis but is present during the middle phases of osteoblast differentiation under osteogenic conditions (Askmyr et al. 2009; Hu et al. 2005), suggesting that the extracellular secretion of collagen XII is dependent on collagen I secretion. Therefore, collagen VI might be secreted earlier than collagen XII, and these two collagens might function together when collagen XII is secreted by osteoblasts during bone formation.

Although mutations in collagens VI or XII cause BM and UCMD, the functional contributions of collagen VI and XII in these diseases are still controversial (Allamand et al. 2011; Bernardi and Bonaldo. 2008; Grumati et al. 2010; Hicks et al. 2014; Lamande et al. 2002; Zou et al. 2014). Dysfunction of mitochondria and impaired clearance by autophagy has been shown to induce spontaneous apoptosis, leading to the development of BM and UCMD (Bonaldo et al. 1998; Grumati et al. 2010; Irwin et al. 2003). Because the disruption of cell-cell connections induces apoptosis (Kalvelyte et al. 2003; Wilson et al. 2000), our data indicate that the impaired formation of communicating cell networks between myocytes and muscular fibroblasts and tenocytes, skin fibroblasts, and osteoblasts might be the key to developing BM and UCMD.

In conclusion, we demonstrate, for the first time, that a functional interaction occurs between collagens VI and XII in primary osteoblasts during osteogenesis. Although collagens VI and XII are different subtypes of collagen, both collagens are indispensable for function in establishing communicating cell networks at bone-forming sites, as required for appropriate bone formation.

## Electronic supplementary material

Below is the link to the electronic supplementary material.

Supplementary Fig. 1 Collagens VI and XII were partially colocalized in osteogenic medium. Immunofluorescent staining of collagens VI (*green*) and XII (*red*) was performed in primary osteoblasts after 2, 4, and 11 days of culture in non-osteogenic medium (**a**) or osteogenic medium (**b**) with permeabilization by Triton X-100. DAPI (*blue*) was used as a nuclear marker. Bars = 50 μm. (**a**) Collagen VI was localized intra- and extracellularly at all stages, whereas collagen XII localization was restricted to the cytoplasm in non-osteogenic medium. (**b**) Immunoreactivity for collagens VI and XII was detected in the extracellular milieu in osteogenic medium. Extracellular collagens VI and XII were localized between adjacent cells, with partial colocalization (*arrows*) on days 2 and 4. On day 11, collagens VI and XII were detected as extracellular microfibril network surrounding individual cells. (GIF 432 kb)

(TIFF 12993 kb)

Supplementary Fig. 2 Collagens I and XII were colocalized pericellularly but not in matrix bridges in osteogenic medium. Immunofluorescent staining of collagens I (*green*) and XII (*red*) was performed in primary osteoblasts after 2, 4, and 11 days of culture in non-osteogenic medium (**a**) or osteogenic medium (**b**) with permeabilization by Triton X-100. DAPI (*blue*) was used as a nuclear marker. Bars = 50 μm. (**a**) Collagens I and XII were localized restricted to cytoplasm. (**b**) Collagens I and XII were localized both intra- and extracellularly in osteogenic medium. Extracellular collagen I was colocalized with collagen XII (*arrows*) pericellularly on days 2 and 4. On day 4, extracellular collagen XII, which was localized between adjacent cells, was not co-localized with collagen I (*arrow heads*). On day 11, collagens I and XII were detected as fiber networks. (GIF 383 kb)

(TIFF 12747 kb)

Supplementary Fig. 3 Western blot analysis of collagens in primary osteoblasts. Western blotting was performed in cell lysates (**a**) and culture media (**b**) from primary osteoblasts cultured in non-osteogenic medium and osteogenic medium. (**a**) Collagens VI, XII, and I were detected in lysates from cells cultured in non-osteogenic medium or osteogenic medium. (**b**) Collagen VI was detected in medium from cells cultured in non-osteogenic medium and osteogenic medium. In contrast, collagen XII was only detected in medium from cells cultured in osteogenic medium. Although collagen I was detected in both non-osteogenic and osteogenic media, the processed form (lower bands) was only detected in medium from cells cultured in osteogenic medium. (GIF 40 kb)

(TIFF 893 kb)
