# Supplementary figures and images for: Collagens VI and XII form complexes mediating osteoblast interactions during osteogenesis
Source: Cell Tissue Res. 2016 Jan 12;364:623–35. doi: 10.1007/s00441-015-2345-y (PMC4875952; doi:10.1007/s00441-015-2345-y)

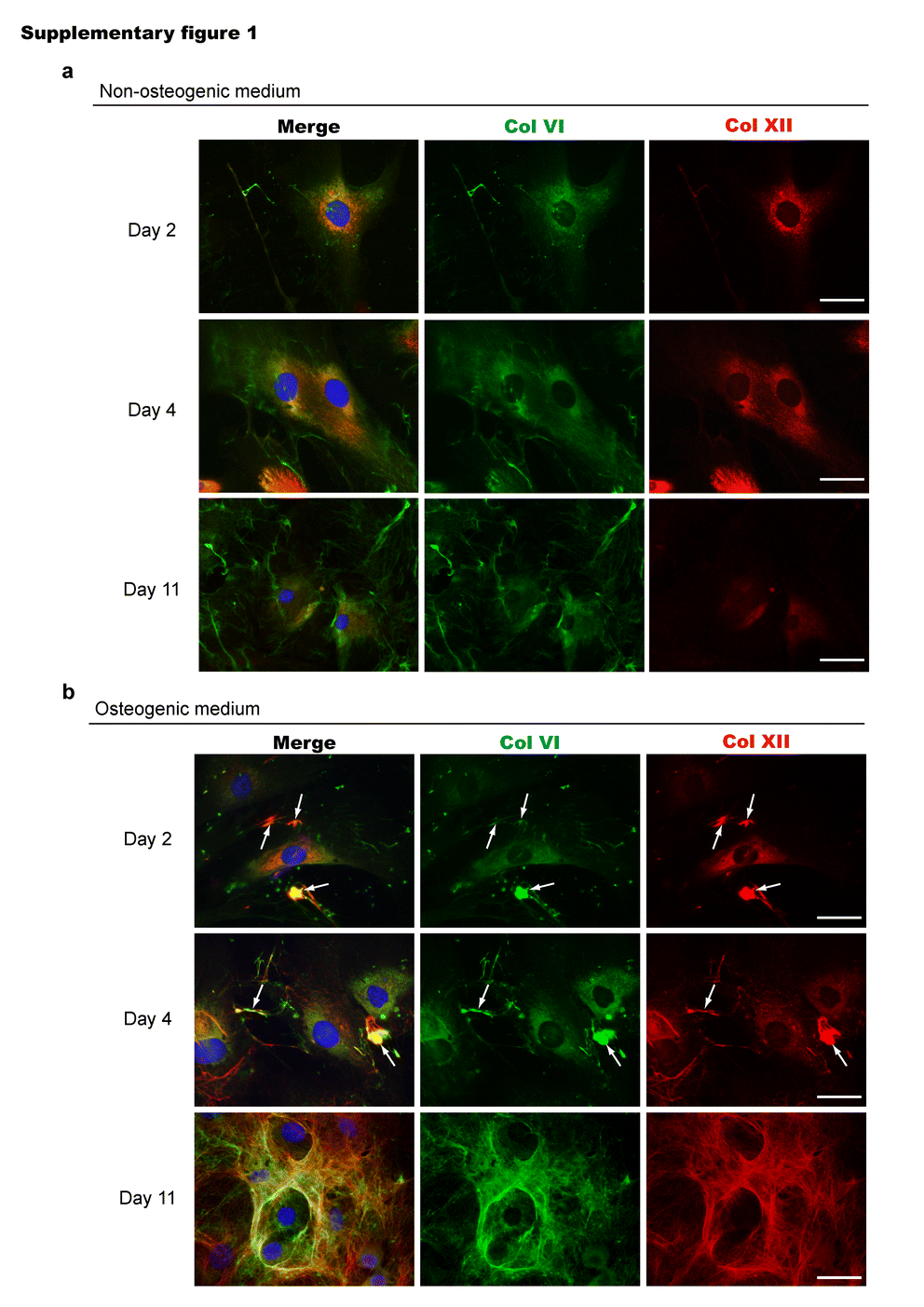

Supplement: Supplementary file 1 — Collagens VI and XII were partially colocalized in osteogenic medium. Immunofluorescent staining of collagens VI (green) and XII (red) was performed in primary osteoblasts after 2, 4, and 11 days of culture in non-osteogenic medium (a) or osteogenic medium (b) with permeabilization by Triton X-100. DAPI (blue) was used as a nuclear marker. Bars = 50 μm. (a) Collagen VI was localized intra- and extracellularly at all stages, whereas collagen XII localization was restricted to the cytoplasm in non-osteogenic medium. (b) Immunoreactivity for collagens VI and XII was detected in the extracellular milieu in osteogenic medium. Extracellular collagens VI and XII were localized between adjacent cells, with partial colocalization (arrows) on days 2 and 4. On day 11, collagens VI and XII were detected as extracellular microfibril network surrounding individual cells. (GIF 432 kb) [file 441_2015_2345_Fig7_ESM.gif]

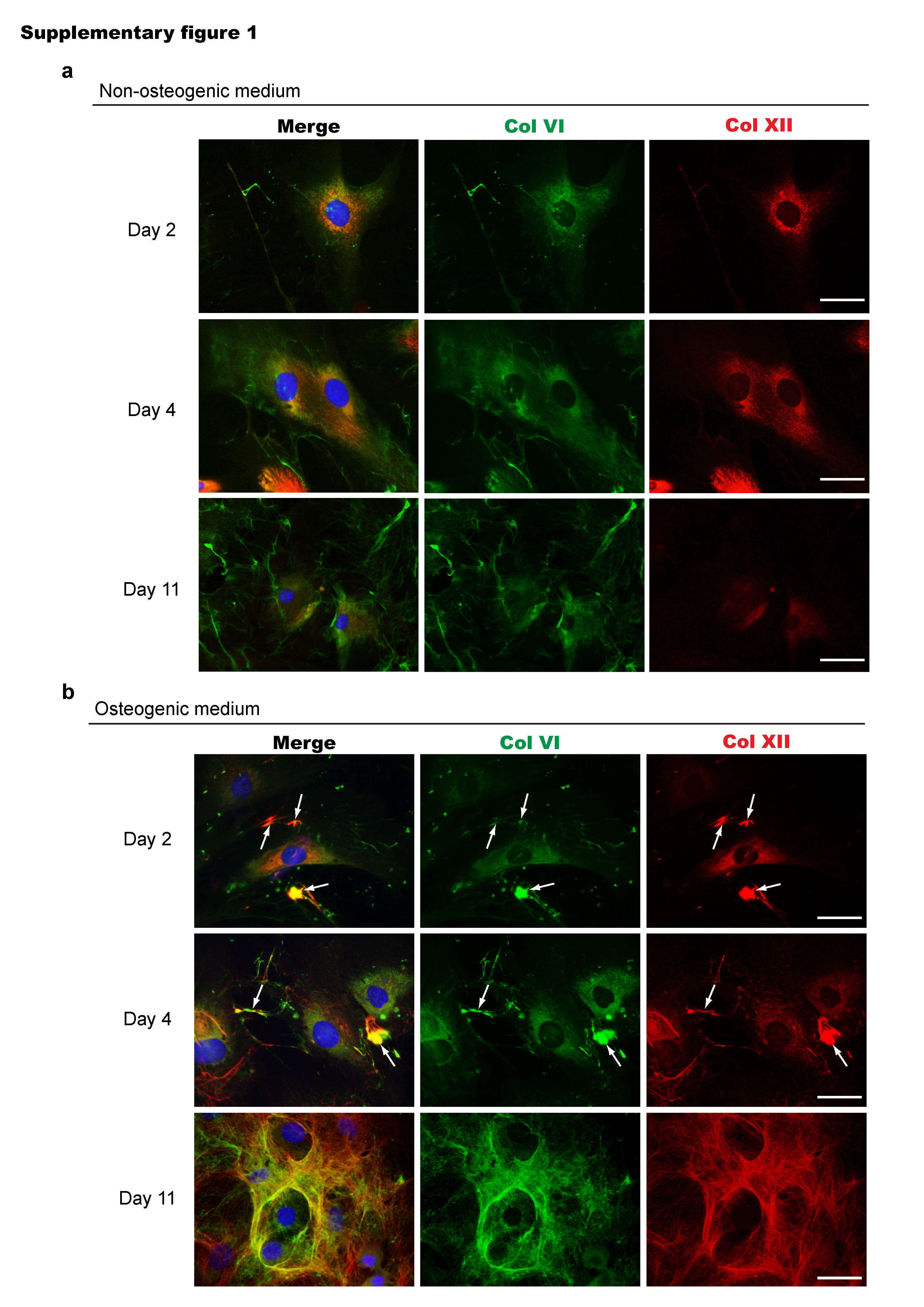

Supplement: Supplementary file 2 — (TIFF 12993 kb) [file 441_2015_2345_MOESM1_ESM.tiff]

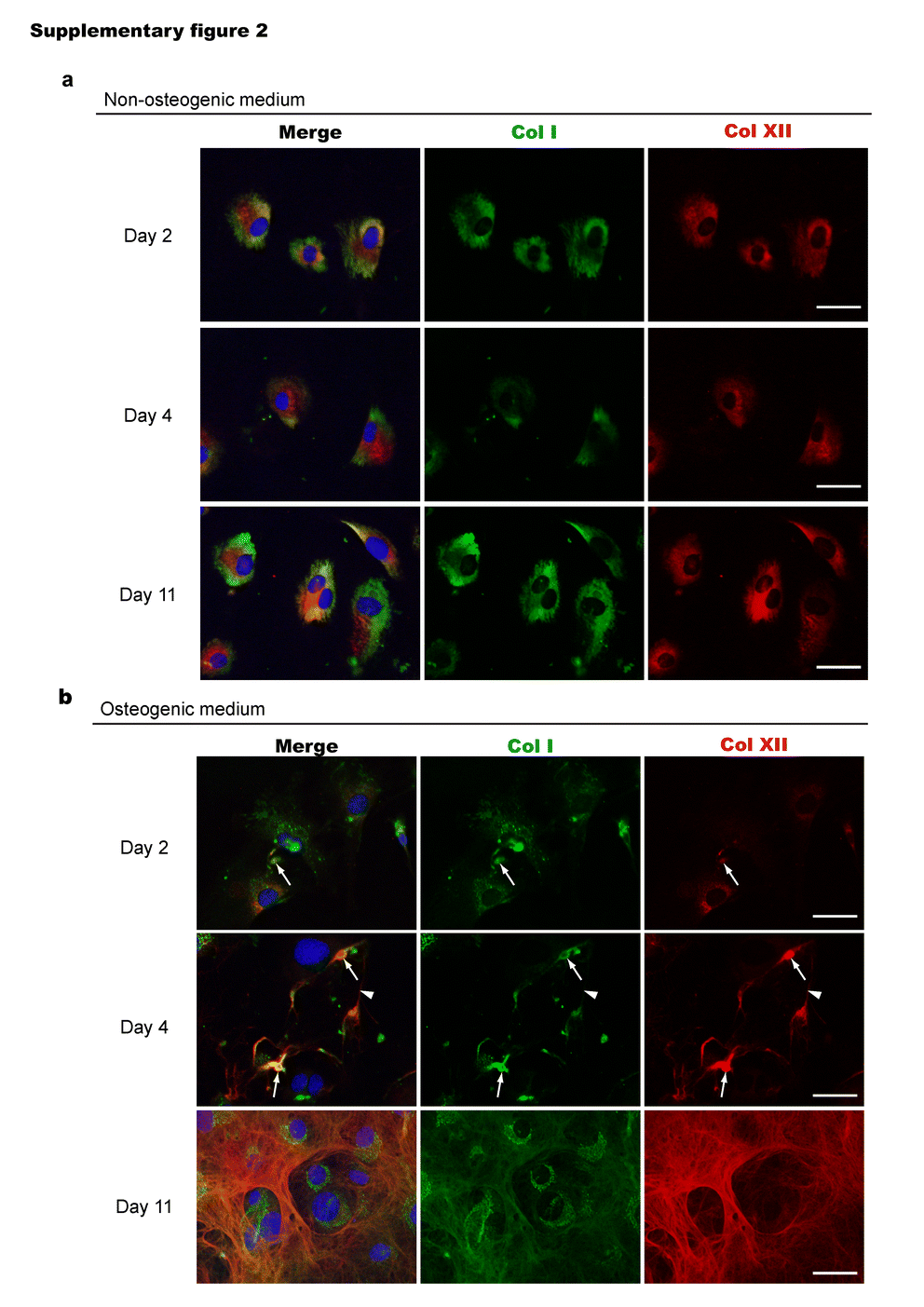

Supplement: Supplementary file 3 — Collagens I and XII were colocalized pericellularly but not in matrix bridges in osteogenic medium. Immunofluorescent staining of collagens I (green) and XII (red) was performed in primary osteoblasts after 2, 4, and 11 days of culture in non-osteogenic medium (a) or osteogenic medium (b) with permeabilization by Triton X-100. DAPI (blue) was used as a nuclear marker. Bars = 50 μm. (a) Collagens I and XII were localized restricted to cytoplasm. (b) Collagens I and XII were localized both intra- and extracellularly in osteogenic medium. Extracellular collagen I was colocalized with collagen XII (arrows) pericellularly on days 2 and 4. On day 4, extracellular collagen XII, which was localized between adjacent cells, was not co-localized with collagen I (arrow heads). On day 11, collagens I and XII were detected as fiber networks. (GIF 383 kb) [file 441_2015_2345_Fig8_ESM.gif]

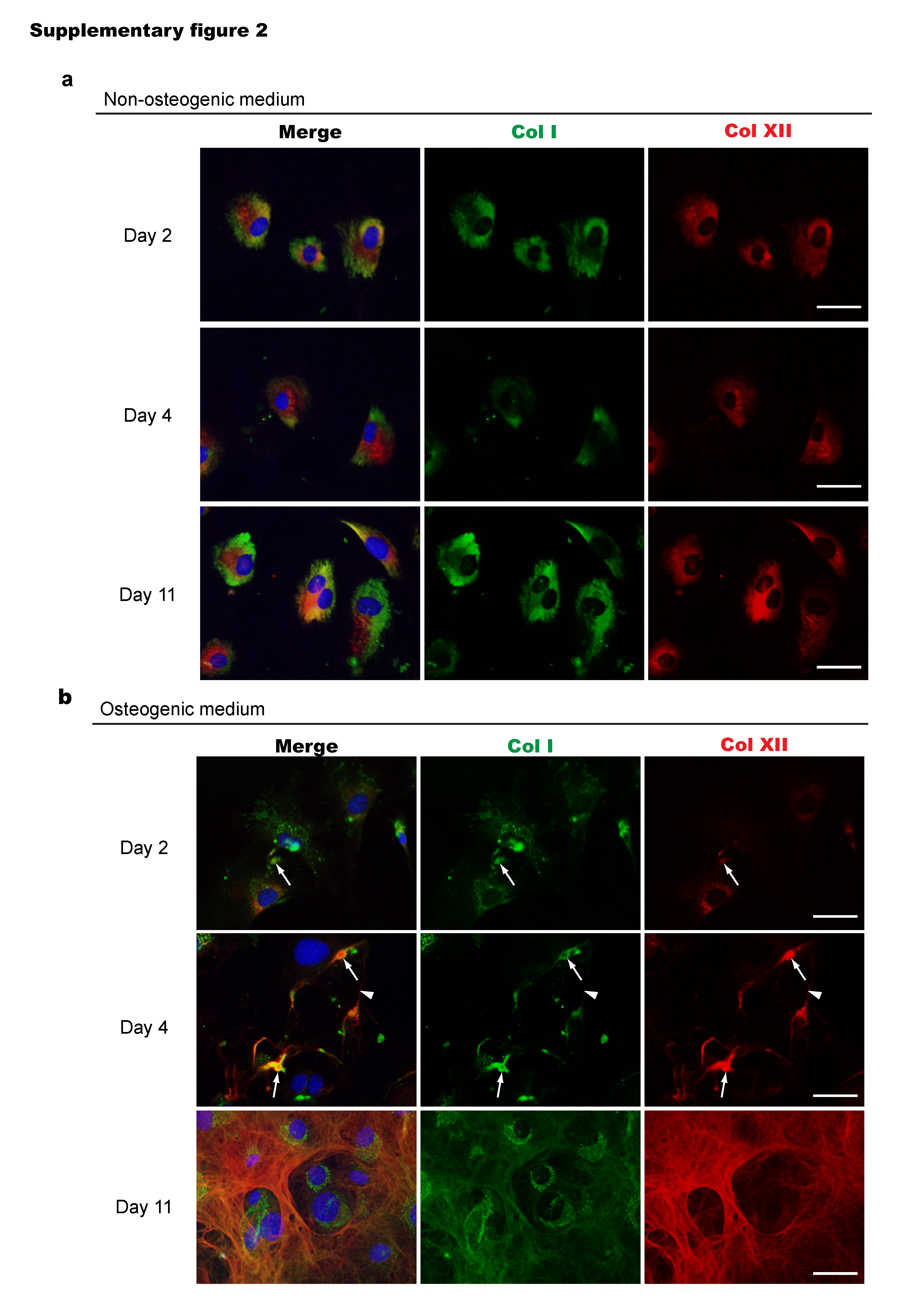

Supplement: Supplementary file 4 — (TIFF 12747 kb) [file 441_2015_2345_MOESM2_ESM.tiff]

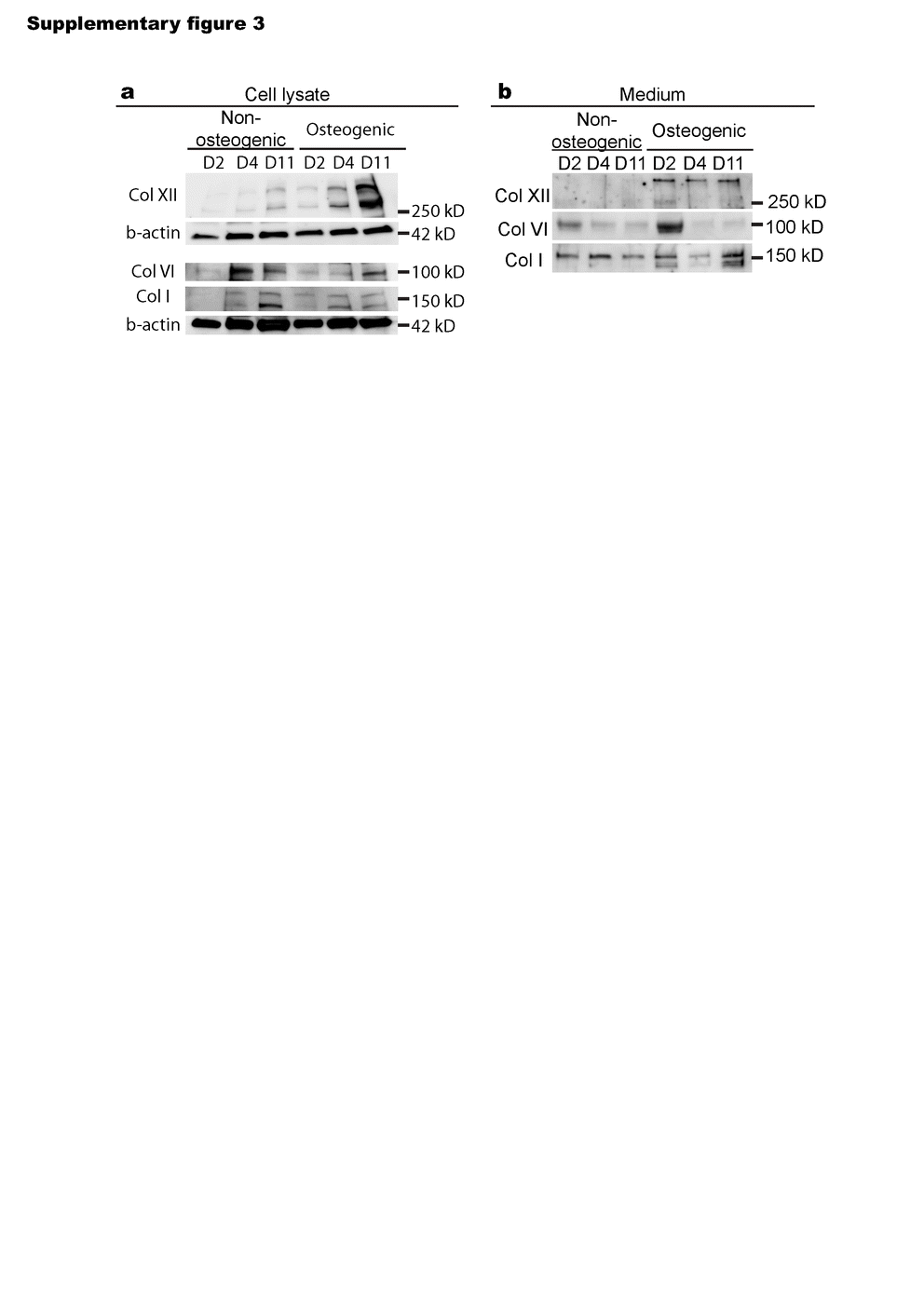

Supplement: Supplementary file 5 — Western blot analysis of collagens in primary osteoblasts. Western blotting was performed in cell lysates (a) and culture media (b) from primary osteoblasts cultured in non-osteogenic medium and osteogenic medium. (a) Collagens VI, XII, and I were detected in lysates from cells cultured in non-osteogenic medium or osteogenic medium. (b) Collagen VI was detected in medium from cells cultured in non-osteogenic medium and osteogenic medium. In contrast, collagen XII was only detected in medium from cells cultured in osteogenic medium. Although collagen I was detected in both non-osteogenic and osteogenic media, the processed form (lower bands) was only detected in medium from cells cultured in osteogenic medium. (GIF 40 kb) [file 441_2015_2345_Fig9_ESM.gif]

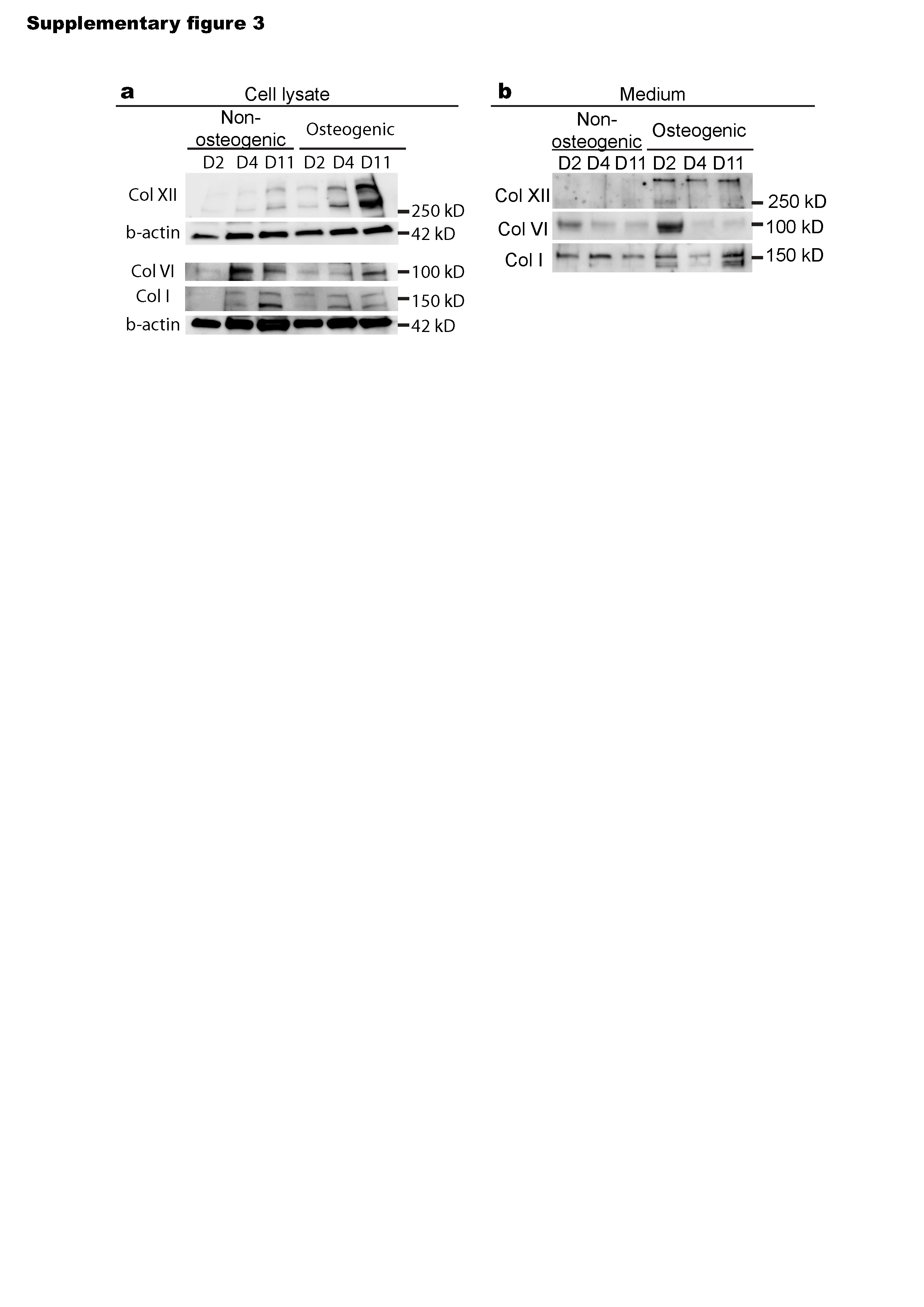

Supplement: Supplementary file 6 — (TIFF 893 kb) [file 441_2015_2345_MOESM3_ESM.tiff]
